# Antifungal Effect of *Bacillus velezensis* ZN-S10 against Plant Pathogen *Colletotrichum changpingense* and Its Inhibition Mechanism

**DOI:** 10.3390/ijms242316694

**Published:** 2023-11-24

**Authors:** Qingling Ye, Zhupeiqi Zhong, Shufeng Chao, Lu Liu, Mengli Chen, Xiaoxiao Feng, Huiming Wu

**Affiliations:** 1 Jixian Honors College, Zhejiang Agriculture and Forestry University, Hangzhou 311300, China; yeql_yan@163.com; 2 College of Advanced Agriculture Sciences, Zhejiang Agriculture and Forestry University, Hangzhou 311300, China; 15869247671@163.com (Z.Z.); chaoshufen@163.com (S.C.); lliulu_2000@163.com (L.L.); cmlmeng@zafu.edu.cn (M.C.); 3 Agricultural Experiment Station, Zhejiang University, Hangzhou 310058, China

**Keywords:** *Bacillus velezensis*, antifungal activity, bioactive metabolites, untargeted metabolomics

## Abstract

In order to optimize crop production and mitigate the adverse impacts associated with the utilization of chemical agents, it is necessary to explore new biocontrol agents. *Bacillus velezensis* has been widely studied as a biocontrol agent because of its efficient and ecofriendly plant disease control mechanisms. This study shows that the strain ZN-S10 effectively reduces the area of leaf spots caused by the pathogen *Colletotrichum changpingense* ZAFU0163-1, which affects conidia production and germination, inhibits mycelium growth, and induces mycelium deformation. In antifungal experiments with crude extracts, we observed a delay in the cell cycle of conidia, which may be responsible for the inhibition of conidial germination. Among the bioactive metabolites detected through integrated LC-MS- and GC-MS-based untargeted metabolomics, 7-O-Succinyl macrolactin A, telocinobufagin, and surfactin A may be the main antifungal metabolites of strain ZN-S10. The presence of 7-O-Succinyl macrolactin A could explain the cell damage in germ tubes. This is the first report of telocinobufagin detected in *B. velezensis*. These results are significant for understanding the inhibitory mechanisms employed by *B. velezensis* and should serve as a reference in the production of biocontrol agents.

## 1. Introduction

The genus *Colletotrichum* comprises numerous globally significant plant pathogens that have a global impact, causing significant losses to economically important crops, especially fruits and vegetables [1]. These pathogens can affect hosts at almost any developmental stage and are capable of causing crown rot disease, leaf spots, and fruit anthracnose lesions [2]. Sunken and necrotic lesions with the formation of tanned- or salmon-colored conidial masses generally appear on the infected area [3]. Strategies for managing anthracnose include using agricultural practices, breeding resistant varieties, and applying chemical fungicides, which play a significant role [4]. However, the excessive application of fungicide may lead to the emergence of resistant pathogens, raising concerns about food safety and environmental pollution [5]. In recent years, there has been increased interest in the biological control of plant diseases as a means to replacing chemical fungicides [6]. Thus far, many bacterial species, such as *Stenotrophomonas rhizophila* [7], *Pseudomonas fluorescens* [8,9], *Streptomyces* sp. [10], and *Bacillus* spp. [11,12], have been recognized as potential biocontrol agents for controlling anthracnose disease.

The antimicrobial effects of *Bacillus* species have been demonstrated in various investigations. They can lessen or prevent the deleterious effects of certain pathogenic organisms through a combination of several mechanisms, including competing for environmental niche and nutrition, stimulating induced systemic resistance (ISR), producing antimicrobial secondary metabolites, and promoting plant growth [13,14,15]. Furthermore, *Bacillus* species are suitable for formulation into stable dry powders with a long shelf life due to the formation of endospores, which can survive heat exposure and desiccation [16]. In addition, it is worth noting that *Bacillus* species are already prevalent in the microflora of plant roots. As a result, plant root microbial communities are unlikely to be negatively impacted [17]. Several *Bacillus*-based products, including Alinit Kodiak (*B. subtilis* GB03), Quantum-400 (*B. subtilis* GB03), Rhizovital (*B. amyloliquefaciens* FZB42), Sonata (*B. pumilus* QST2808, and YIB (*Bacillus* spp.), are commercially available [18,19].

*B. velezensis*, as an active biocontrol bacterium, can produce a vast array of bioactive metabolites such as lipopeptides (i.e., surfactin, bacillomycin-D, fengycin, and bacillibactin), polyketide-type antimicrobial molecules (i.e., macrolactin, bacillaene, and difficidin), and other beneficial metabolites (i.e., siderophores, bacteriocins, and volatile organic compounds (VOCs)) [17]. With these metabolites, *B. velezensis* can resist pathogenic organisms (i.e., bacteria, fungi, nematodes, viruses, and insects), promote plant growth, and induce ISR [20]. A large number of experiments have provided substantial evidence showcasing the inhibitory impact of *B. velezensis* on various types of fungi, including *Fusarium solani*, *Candida albicans,* and *Colletotrichum* spp. [20,21,22,23,24]. It can be seen that *B. velezensis* is an important material for the development of new biocontrol agents and the understanding of the plant disease control mechanisms of biocontrol bacterium.

Clarification of the chemical compositions of the metabolites of *B. velezensis* is a key step in in the study of its mechanism. Under a given set of conditions, untargeted metabolomics can detect all the small-molecule metabolites present within a biological system without bias [25]. Differential metabolites, the metabolites that show different concentrations between test and control groups of samples, can be screened through bioinformatics analysis [26]. Gas chromatography-mass spectrometry (GC-MS) and liquid chromatography-mass spectrometry (LC-MS) have been used extensively in untargeted metabolomics [27]. GC-MS analysis contains power capacities of high reproducibility, dynamic range, and a universal mass spectral library for compounds with small molecular weight, such as sugars, organic acids, fatty acids, and amino acids [28]. LC-MS, on the other hand, covers large hydrophobic metabolites predominant in secondary compounds such as alkaloids, terpenoids, and phenols [29]. However, untargeted metabolomics can only show changes in the relative levels, and it is not possible to quantify them due to the larger number of variables and because the identity of the metabolites is often unknown [30].

In this study, we investigated the antifungal effect of *Bacillus velezensis* ZN-S10 on an anthracnose pathogen, *Colletotrichum changpingense* ZAFU0163-1, through in vivo and in vitro experiments. Antifungal experiments using crude extracts were conducted to observe the cell cycle of spores. Additionally, LC-MS and GC-MS untargeted metabolomics were used to screen for the major antifungal metabolites of ZN-S10, with the aim of providing inspiration for the further research and development of scientific and environmental control strategies for this disease.

## 2. Results

### 2.1. Antagonistic Activity of B. velezensis ZN-S10 against Phytopathogenic Fungi

**Figure 1:**
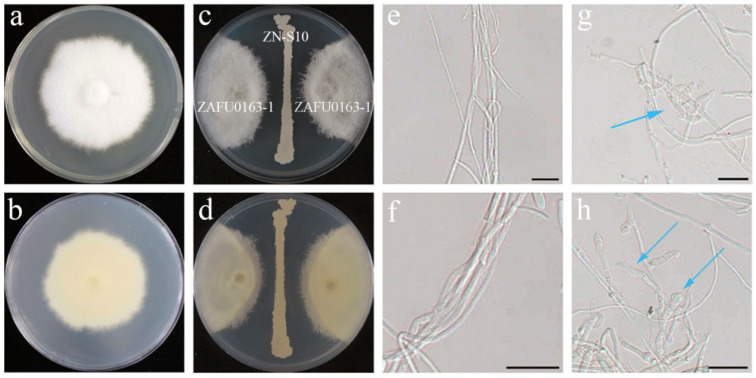
Antagonistic activity of *B. velezensis* ZN-S10 against *C. changpingense* ZAFU0163-1. (**a**–**d**) Antagonistic activity of ZN-S10 on WA plates at 25 °C for 5 days: (**a**) the control group, (**c**) treatment group, and (**b**,**d**) the backside of (**a**,**b**), respectively. (**e**–**h**) Morphology of affected mycelia of strain ZAFU0163-1: the blue arrows in (**g**,**h**) respectively represent the curled and twisted mycelia and irregular expanded mycelia in the treatment group compared with the control group (**e**,**f**). (Scale bar = 20 µm).

A dual-culture assay was carried out to evaluate the impact of ZN-S10 on the fungal pathogen *Colletotrichum changpingense* ZAFU0163-1 on Waksman’s agar (WA) plates, incubated at 25 °C for 5 days; four replications were considered for each treatment. Results showed that ZN-S10 restrained the growth of the fungal pathogen strain ZAFU0163-1 (Figure 1c,d). Under microscopic observation, the mycelia affected by ZN-S10 showed obvious deformities compared to the control group, including irregular expansion and curled, twisted mycelium, (Figure 1g,h).

### 2.2. Detached Leaf Inoculation Test

**Figure 2:**
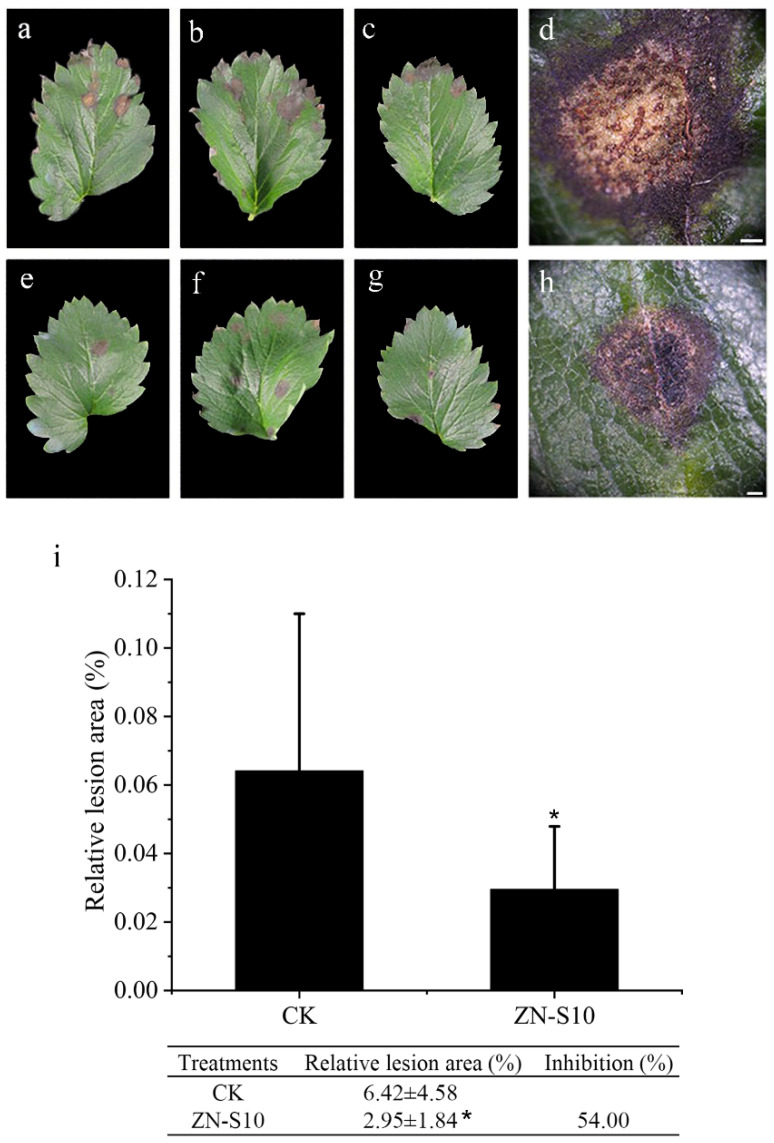
Pathogenicity assay of *C. changpingense* ZAFU0163-1 on strawberry leaves. Symptoms on untreated (**a**–**d**) and treated (**e**–**h**) leaves after 5 d of inoculation. Disease severity (**i**). Data are means of replicates with ± standard deviation (SD). Asterisks represent differences compared with CK ac-cording to the LSD test (* *p* ≤ 0.05) and have been reported in the graph. (Scale bar = 500 µm).

Five days after inoculation at 30 °C, all the leaves—both those treated with sterilized water and 10^8^ CFU/mL of *B. velezensis* ZN-S10 bacterial suspension—that had been exposed to the pathogen exhibited symptoms of disease, but the relative area of disease in the experimental group was significantly smaller than that in the control group (*p*-value ≤ 0.05); in other words the disease severity was lower. The impact of ZAFU0163-1 on the development of leaf spots after bacterial suspension inoculation revealed that ZN-S10 reduced disease severity by 54.00% compared to the control group (CK). In the CK, there were visible auburn spore masses on the lesions (Figure 2g), whereas no such masses were observed in the treated group (Figure 2h).

### 2.3. Inhibitory Effect of Volatile Organic Compounds (VOCs) Released by B. velezensis ZN-S10 on the Growth of C. changpingense ZAFU0163-1

**Figure 3:**
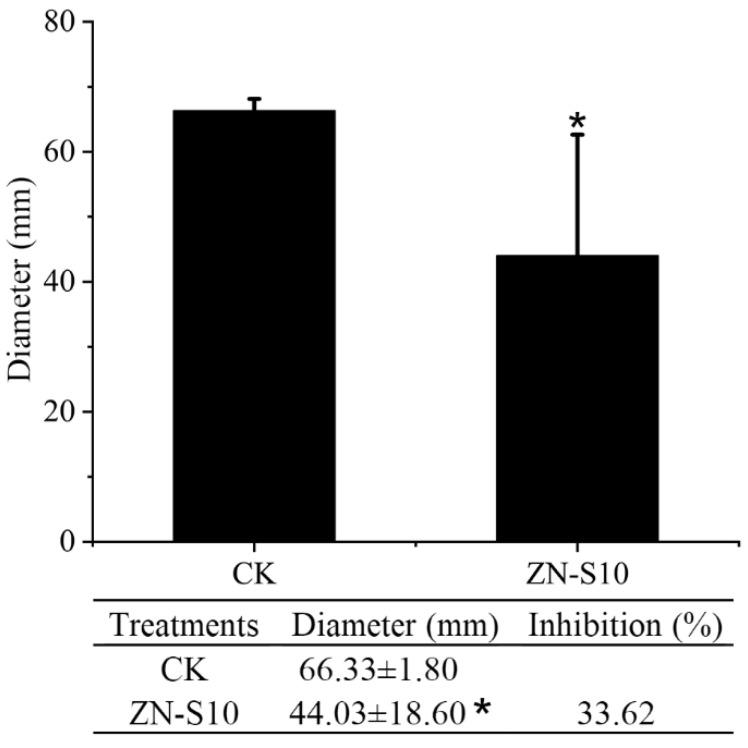
Inhibition of mycelial growth of *C. changpingense* ZAFU0163-1 by volatile organic compounds produced by *B. velezensis* ZN-S10. Asterisks represent differences compared with CK according to the LSD test (* *p* ≤ 0.05) and have been reported in the graph. Data are means of replicates with ± standard deviation (SD).

The VOCs produced by ZN-S10 affected the mycelial growth of ZAFU0163-1, showing a significant effect, with a low *p*-value (≤0.05) (Figure 3). Plates spread with ZN-S10 and fungal plates were sealed together facing each other; when incubated at 25 °C for 5 days, the inhibitory effect of these VOCs was observed as 33.62%. This finding indicates that ZN-S10 possesses the ability to influence the growth dynamics of ZAFU0163-1 through the release of bioactive VOCs.

### 2.4. Nonvolatile Compound Antifungal Test on Hyphal and Conidial Germination

**Figure 4:**
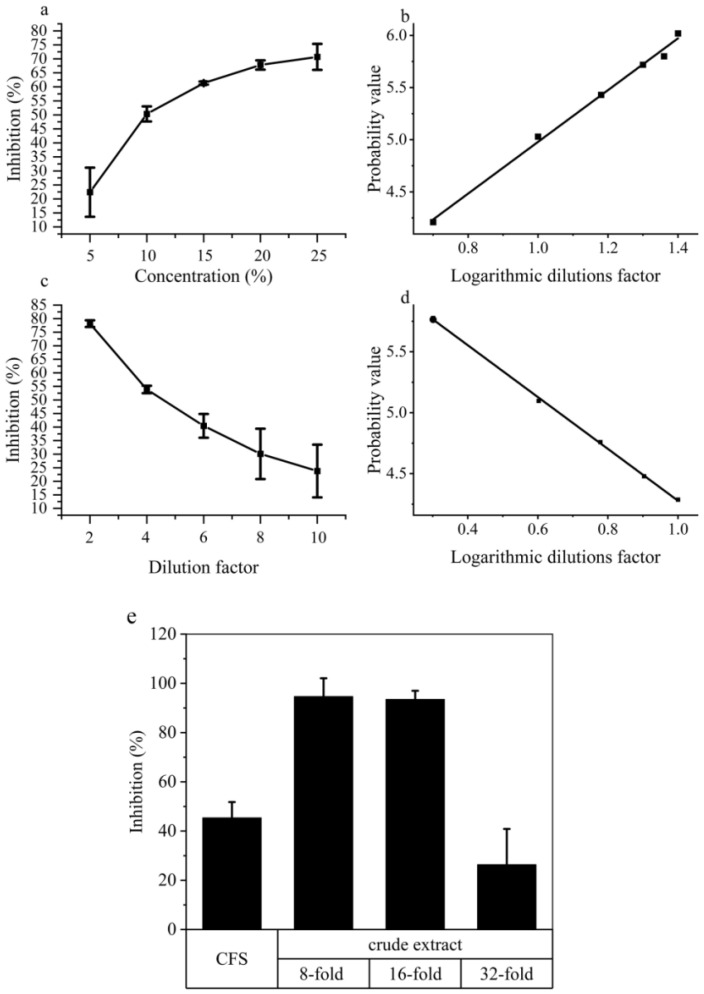
Antifungal effect of cell-free synthesis (CFS) and C18 solid phase extraction (SPE) crude extract of *B. velezensis* ZN-S10. Inhibition of mycelial growth of (**a**) CFS and (**c**) crude extract demonstrated the antifungal activity of ZN-S10 against ZAFU0163-1. The EC_50_ values are expressed as the activity of CFS concentration (**b**) and crude extract dilution factor (**d**). Inhibition of conidial germination of CFS and crude extract are shown in (**e**).

**Figure 5:**
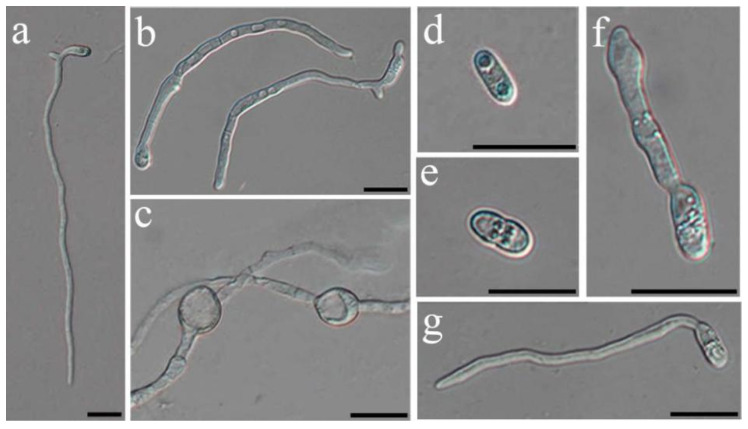
Light micrographs for conidial germination of the inhibitory effect of cell-free synthesis and C18 solid phase extraction (SPE) crude extract of *B. velezensis* ZN-S10. Cell damage (**b**) and swollen cells (**c**) were present in the CFS group as compared to the LB group (**a**). The delayed conidial germination process due to the exposure to the crude extract ((**d**): 8-fold; (**e**): 16-fold; (**f**): 32-fold) compared with methanol group (**g**). (Scale bar = 20 µm).

The nonvolatile metabolite test demonstrated a significant increase in the inhibitory effect on mycelial growth as the concentration increased. The EC_50_ (concentration for 50% of maximal effect) of cell-free synthesis (CFS) on Potato Dextrose Agar (PDA) medium was 11.68% (mL/mL) (Figure 4b). Even after a tenfold dilution, the C18 solid phase extraction (SPE) crude extract still retained a 30.1% inhibitory activity, and its EC_50_ was determined to be 0.22% (mL/mL) after a 4.6-fold dilution (Figure 4c,d). Furthermore, both the CFS and the crude extract of ZN-S10 significantly affected the conidial germination, yielding a *p*-value ≤ 0.01 compared with the LB and methanol groups, respectively (Figure S1). The CFS inhibited 45.39% of the spore germination, induced cell damage, and caused cell swelling when compared to the control group (Figure 4e and Figure 5a–c). Microscopic observation revealed that the conidial germination process might be delayed due to exposure to the crude extract of ZN-S10 (Figure 5d–g), with the highest inhibition exceeding 90% (Figure 4e).

### 2.5. Metabolic Profiles Analyzed Using Untargeted Metabolomics

Integrated liquid-chromatography-mass-spectrometry- (LC-MS) and gas-chromatography-mass spectrometry (GC-MS)-based untargeted metabolomics were used to analyze metabolites of ZN-S10. A total of 1185 metabolites were detected in LC-MS and 338 in GC-MS. Principal component analysis (PCA) was employed to evaluate the quality of the data from LC-MS and GC-MS. The results indicated that the samples in the experimental group were closely clustered together without discrete points generated, confirming that the experimental samples were parallel and that the data were reliable. There was no intersection between the samples in the experimental and control groups (CK), indicating that the main components of the crude extract in the experimental and blank groups were different (Figure S2).

**Figure 6:**
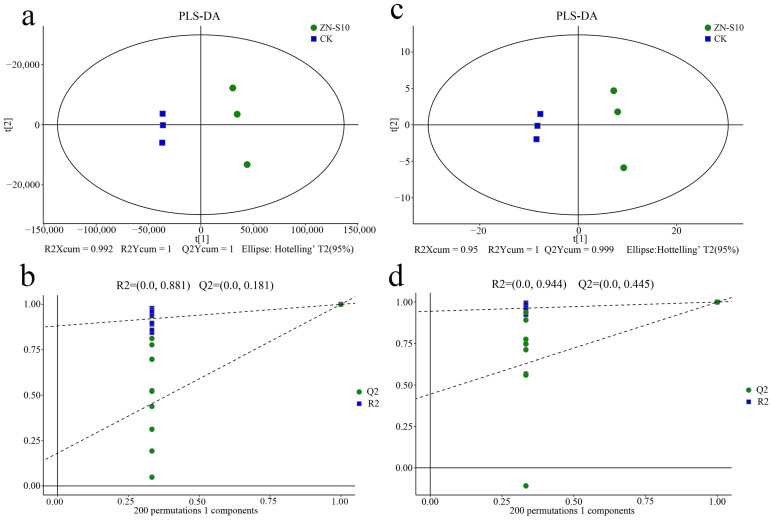
PLS-DA score plots with corresponding permutation test plots derived from the (**a**,**b**) LC–MS and (**c**,**d**) GC–MS metabolite profiles. (**b**,**c**) Permutation test plots for PLS-DA score plots (**a**) and (**c**), respectively. The permutation tests were carried out with 200 random permutations.

Supervised partial least squares discrimination analysis (PLS-DA) was performed to identify the metabolites responsible for the separation between the CK and ZN-S10 groups. Sevenfold internal cross-validation and a 200-time permutation test were further conducted to evaluate the predictive accuracy and statistical significance of these models. In the LC-MS analysis, the predictive accuracy parameters of the PLS-DA model were R2Xcum = 0.992, R2Ycum = 1, and Q2Ycum = 1 (Figure 6a), with their corresponding statistical significance being R2 = 0.881 and Q2 = 0.181 (Figure 6b). In the GC-MS analysis, the predictive accuracy parameters of the PLS-DA model were R2Xcum = 0.95, R2Ycum = 1, and Q2Ycum = 0.999 (Figure 6c), with their corresponding statistical significance being R2 = 0.944 and Q2 = 0.445 (Figure 6d). These PLS-DA models exhibited a low risk of overfitting based on the criterion that the green Q2 values to the left were all lower than the original points to the right (Figure 6b,d). These findings indicated that these PLS-DA models could identify the differentially enriched metabolites between the CK and ZN-S10 groups.

### 2.6. Differential Metabolites in the Crude Extract Compounds

A total of 57 differential metabolites were selected in the LC-MS and 37 differential metabolites in the GC-MS using the selected screening criteria (VIP values > 1.5 and *q* values < 0.01) (Figure S3). In order to identify the main antifungal substances, the selected screening criteria fold changes (FC) > 1000 and FC > 2.0 were respectively introduced in the LC-MS and GC–MS analyses. A total of 19 substances were screened from the differential metabolites in LC-MS (Table 1) and 23 differential metabolites in GC-MS (Table 2). Fatty acids were the most common, with nine in total, and were followed by five steroids and steroid derivatives; four organooxygen compounds and organonitrogen compounds; two each of macrolides, glycerophospholipids, acids, imidazopyrimidines, and purine nucleotides’ and finally one each of peptidomimetics, prenol lipids, polyketides, benzene and substituted derivatives, keto acids and derivatives, organic carbonic acids and derivatives, organic phosphoric acids and derivatives, diazines, and pteridines and derivatives; and three unclassified (5-Methoxy-1-Pentanol, Hydrobenzoin, and 4-Nitrophenethylamine).

## 3. Discussion

Generally, *Colletotrichum* spp. penetrate and colonize hosts through spore germination and appressorium formation. They can also directly infect hosts through stomata and wounds using hyphae [48]. Upon infection, secondary hyphae invade the surrounding cells, causing necrotrophic anthracnose symptoms. The pathogens can continue to produce new conidia throughout the season, resulting in a polycyclic disease cycle [49]. Previous reports have shown that *Bacillus velezensis* can inhibit the growth of hyphae and the germination of conidia, effectively combating fungal pathogens [50,51,52]. This study demonstrates that ZN-S10 can successfully control anthracnose development in strawberry plants and influence the production of new conidia (Figure 2). In dual-culture tests, ZN-S10 strongly inhibited mycelial pathogen growth and caused hyphal alterations (Figure 1), which is consistent with previous findings. Similarly, volatile organic compounds (VOCs) produced by ZN-S10 were effective in regulating mycelial growth (Figure 3). The cell-free synthesis (CFS) and crude extract of ZN-S10 both exerted a significant inhibitory effect on conidial germination and mycelial growth. The crude extract may have caused a delay in the cell cycle of pathogenic conidia, resulting in the reduced germination rate.

Appressorium formation plays a crucial role in the development of anthracnose disease in this pathosystem. Research has illustrated that the *B. velezensis* metabolite can inhibit appressorium formation [53]. However, appressoria of *C. changpingense* have not been observed [54]. To provide a better understanding of the interaction between ZN-S10 and *Colletotrichum*, it would be interesting to elucidate whether the formation of appressoria is affected during their interaction.

In vitro and in vivo tests indicated that both secreted metabolites and VOCs of strain ZN-S10 could act as biocontrol agents in counteracting plant pathogens, establishing the research value of ZN-S10 as a potential biological agent. The results of LC-MS analysis showed that ZN-S10 produced 19 main differentially abundant metabolites, with 7-O-Succinyl macrolactin A, telocinobufagin, and surfactin A being the main antifungal active substance according to the variable importance for projection (VIP) and fold change (Table 1). These three metabolites have been identified as antifungal agents in various studies, suggesting their role as the main contributors to the antifungal effect of ZN-S10 [55,56]. Previous studies have proven that all three of these metabolites can have an influence on the growth of hyphae [17,27,57]. Notably, 7-O-Succinyl macrolactin A has been found to cause significant cell damage, which may explain the cell damage on germ tubes in this study [47]. Telocinobufagin has been demonstrated to exert antifungal activity in *Batrachochytrium dendrobatidis*, an amphibian fungal pathogen, indicating its possible use as an antifungal agent [31]. This is the first report on telocinobufagin as a natural product derived from bacteria. Thus, targeted metabolomics should be considered to explore a more comprehensive effect of the differentially abundant metabolites produced by ZN-S10.

Bacterial VOCs can affect the fungal growth, development, differentiation, and production of secondary metabolites [58]. The main VOCs produced by *B. velezensis* include alkenes, alcohols, ketones, terpenes, benzoids, pyrazines, acids, and esters [59]. For example, 2,3-butanedione has the ability to inhibit *Alternaria iridiaustralis*, a pathogenic fungus responsible for rotting wolfberry fruit [60]. In this study, GC-MS analysis of the volatile fraction from the strain ZN-S10 revealed the upregulation of a total of 23 metabolites. Caproic acid, 2,3-butanediol, and urea have been identified as active antifungal compounds [17,43,45,47]. Additionally, pterin and dihydrouracil, with antibacterial and nematocidal activities, were also detected [45,46,47]. These metabolites suggest that ZN-S10 has the potential to act against multiple plant pathogens. The GC-MS analysis also detected molecules related to ISR, plant growth, and biofilm formation.

In summary, we investigated the antifungal ability of the ZN-S10 strain against *C. changpingense*. This was primarily achieved using in vitro and in vivo tests, which revealed that the inhibition of mycelial growth could cause mycelial deformity and inhibition of spore germination. A delay in the cell cycle of conidia may be responsible for inhibiting spore germination. Additionally, we found that strain ZN-S10 produces several secondary metabolites, including 7-O-Succinyl macrolactin A, telocinobufagin, and surfactin A, which contribute to its antifungal activity. These metabolites were successfully obtained with C18-SPE. Using LC-MS and GC-MS analysis, we also detected previously confirmed antifungal and antibacterial metabolites. Meanwhile, the presence of plant growth regulators suggests that ZN-S10 has the potential to regulate various processes in hosts, such as plant growth, stress response, and defense. However, this study only investigated changes in the relative amounts of differential metabolites and did not quantify the differential metabolites. To further investigate their function in plant pathogen resistance, targeted metabolomics needs to be introduced to quantify the major differential metabolites, and these metabolites need to be purified to validate their antifungal effects.

## 4. Materials and Methods

### 4.1. Culture Conditions for Bacterial and Fungal Strains

The antagonistic bacterial *Bacillus velezensis* ZN-S10 (NCBI BioSample ID SAMN30381276) was isolated from tomato roots. Strain ZN-S10 was subcultured onto lysogenic broth (LB) agar medium, and a 24-h-old single colony was randomly picked for incubation in LB liquid medium at 28 °C and 180 rpm overnight in a shaking incubator. The fungal pathogen *Colletotrichum changpingense* ZAFU0163-1 was isolated from strawberry stems and subcultured on Potato Dextrose Agar (PDA) medium at 25 °C for 5 days [54].

### 4.2. Antagonistic Activity of B. velezensis ZN-S10 against Colletotrichum

#### 4.2.1. Dual-Culture Assay

A dual-culture assay was introduced to evaluate the antagonistic activity of strain ZN-S10 against ZAFU0163-1. One loopful colony of ZN-S10 was streaked on the center of a Waksman’s agar (WA) plate, with a 5 mm diameter mycelial plug of 5-day-old ZAFU0163-1 placed on each side. The WA medium inoculated with pathogen plugs alone was used as a control, and four replications were considered for each treatment. The plates were incubated at 25 °C for 5 days, and hyphal abnormalities caused by ZN-S10 were observed under a light microscope.

#### 4.2.2. Detached Leaves Inoculation

The spore suspension was prepared using the *C. changpingense* ZAFU0163-1 spore-producing PDA plates. The surface of fully sporulated fungal colony was flooded with 10 mL of sterile water and gently scrubbed with spreader. The fungal suspension was filtered through sterile triple-lens-cleaning tissues to remove mycelia and adjusted to 1 × 10^5^ CFU/mL using a hemocytometer cell-counting chamber. One-day-old preinoculated broth culture of *B. velezensis* ZN-S10 was centrifuged at 1699× *g* for 10 min and then resuspended to 1 × 10^8^ CFU/mL with sterilize water.

Healthy strawberry leaves were used for the pathogenicity test. Leaves were surface-sterilized with 75% ethanol and washed three times with distilled water. Fifteen leaves were uniformly sprayed with 24 h old bacterial suspension (1 × 10^8^ CFU/mL) of ZN-S10. Control leaves were inoculated with sterilized water. Conidial suspension was applied to all leaves, and they were then placed into a plastic box covered with plastic film and incubated at 25 ℃ for 7 d. The disease severity was represented by relative lesion area, which was obtained by calculating the ratio of lesion-spot pixels to whole-leaf pixels, and the inhibition was calculated with following equation: inhibition (%) = [(Lc − Lt)/Lc] × 100, where Lc is the relative lesion area of control, and Lt is the relative lesion area of treatment plate.

#### 4.2.3. Volatile Organic Compound (VOC) Inhibition Assay

The volatile antifungal test was performed using the double-plate method. Ten microliters of 24 h old ZN-S10 was spread on an LB medium, and a mycelial disc (5 mm diameter) from a 5-day-old culture of ZAFU0163-1 was inoculated on the PDA plate. Then, the plate of pathogen was inverted on a plate containing ZN-S10 and sealed tightly using parafilm. The plates with the pathogen alone were inoculated as the control. Four replications were carried out for each treatment, and the results investigated after the fungal pathogen colony in the control extended to more than two-thirds of the plate. Mycelial growth inhibition was calculated with the following equation: mycelial growth inhibition (%) = [(D − d)/D] × 100, where D is the diameter of the fungal colony in the control plate, and d is the diameter of the fungal colony in the treatment plate [61].

#### 4.2.4. Nonvolatile Compound Antifungal Test on Hyphal and Conidial Germination

A single colony of ZN-S10 was inoculated in LB liquid medium at 28 °C and 180 rpm for one night. Then, the inoculated culture broth of ZN-S10 was centrifuged at 1699× *g* for 10 min, resuspended to 1 × 10^8^ CFU/mL with sterilized water, and inoculated at 2% (*v*/*v*) into fresh LB liquid medium at 28 °C and 180 rpm for 24 h. After centrifugation (10 min, 1699× *g*) the preinoculated LB culture supernatant of ZN-S10 was collected and filtered through 0.22 μm millipore filter membrane. Cell-free synthesis (CFS) was added to sterile cool WA medium to make different concentrations (5%, 10%, 15%, 20%, and 25%). WA medium with the same concentrations of LB liquid medium was used as the control. A 5 mm diameter mycelial plug of 5-day-old fungal pathogen ZAFU0163-1 was placed at the center of WA plates; all concentrations were repeated four times and incubated at 25 °C for 5 days. The inhibition percentage of mycelial growth of the pathogen was measured using the formula described in Section 4.2.3, and the concentration for 50% of maximal effect (EC_50_) was calculated relative to the control using the Data Processing System (DPS v13.01, Zhejiang University, Hangzhou, China).

The effect of CFS on the conidial germination of the pathogen was examined using 100 μL of spore suspension (1 × 10^6^ CFU/mL) and 100 μL of CFS in a sterile tube containing 800 μL of PDB medium. As a control, a tube with 100 μL of LB liquid medium was used instead of the supernatant. Subsequently, a pipette was used to transfer 50 μL from each tube to a concave microscope slide; four replications were used for each treatment. The slides were incubated at 25 °C for 3 h to calculate conidial germination, germination inhibition (%) was calculated based on the control, and the intensity of germ tube alteration was observed after 7 h. The inhibition percentage of conidial germination was measured according to the following formula: inhibition (%) = [(Pc − Pt)/Pc] × 100, where Pc is the conidial germination percentage of control, and Pt is the conidial germination percentage of treatment.

#### 4.2.5. Extraction of Crude Compounds from the Broth Culture of *B. velezensis* ZN-10 Using C18 Solid Phase Extraction (SPE) and Inhibition Assay

Bacterial CFS was preconcentrated with SPE using 500 mg/6 mL of Cleanert C18-SPE (Agela Technologies, Tianjin, China). The C18-SPE columns were pretreated with 5 mL of sterile water followed by 3 mL of methanol. Excess methanol was removed using sterile water. Then, 100  mL of 24 h CFS was loaded onto the cartridge. Methanol (2 mL, Taicang Hushi Reagent Co., Ltd., Shanghai, China) was used to elute the analytes from the extraction column.

The crude extract was diluted 2, 4, 6, 8, and 10 times, with 1 mL of each being added to 100 mL of sterile cool PDA medium. All plates were inoculated with a mycelial plug (5 mm in diameter) of ZAFU0163-1, and same the plates loaded with 1 mL of methanol were used as controls. Each treatment was replicated four times and inoculated at 25 °C for 5 days. The growth inhibition (%) and EC_50_ were measured using the formula described in Section 4.2.4.

The effect of crude extract on the conidial germination of the pathogen was examined as described in the nonvolatile compound antifungal test. The diluted crude extract was used, with methanol serving as the control.

### 4.3. LC-MS and GC-MS Analyses

An ACQUITY UPLC I-Class plus (Waters, Shanghai, China) coupled with a QE plus (Thermo Fisher Scientific, Shanghai, China) high-resolution tandem mass spectrometer was used for LC-MS analysis. The injection volume was 2 μL. Separation was performed on an Waters ACQUITY UPLC HSS T3 (100 mm × 2.1 mm, 1.8 μm). The column oven was maintained at 45 °C. The mobile phase consisted of solvents A (water + 0.1% formic acid) and B (acetonitrile), with a flow rate of 0.35 mL/min. Formic acid and acetonitrile were provided by Thermo Fisher Scientific. The gradient elution program was set as follows: 0–2 min, 95% A, 5% B; 2–14 min, 95% to 0% A, 5% to 100% B; and 15.1–16 min, 5% A,100% B. The Q-TOF mass spectrometer was operated in both positive and negative ion modes.

For the GC-MS analysis, 1 μL of crude compound was subjected to an Agilent 7890B-5977B GC–MS system. The analysis was performed in splitless mode using a DB-5MS capillary column (30 m × 0.25 mm × 0.25 μm, Agilent J&W Scientific, Folsom, CA, USA). First, helium was used as the carrier gas at a constant flow rate of 1.0 mL/min. Second, the instrument was kept at 60 °C for 0.5 min, ramped to 125 °C at a rate of 8 °C/min, ramped to 210 °C at a rate of 8 °C/min, ramped to 270 °C at a rate of 15 °C/min, and ramped to 305 °C at a rate of 20 °C/min for 5 min. Solvent delay was set at 5 min.

The original LC-MS data were analyzed using Progenesis QI v3.0 (Nonlinear Dynamics, Newcastle, UK) for baseline filtering, peak identification, integration, retention time correction, peak alignment, and normalization. The Human Metabolome Database (HMDB), Lipidmaps (v2.3), and METLIN databases as well as self-constructed libraries were used for identification. Any peaks with a missing value (ion intensity = 0) in more than 50% samples were removed from the dataset. If the total match factor was greater than 50 (full score is 80), then the metabolite identification was reliable.

The original GC-MS data were analyzed using MS-DIAL and identified via comparison to the Untarget GC-MS database from Lumingbio (Shanghai luming biological technology co.Ltd, Shanghai, China). The internal standard was used for data quality control. For internal standards, any known pseudopositive peaks, such as peaks caused by noise, column bleed, and BSTFA derivatization procedure, as well as any peaks with a missing value (ion intensity = 0) in more than 50% samples, were removed from the dataset. The data were normalized using the sum intensity of the peaks in each sample. If the total match factor was greater than 80 (full score is 100), then the metabolite identification was reliable.

### 4.4. Differential Metabolites Analysis

Bioinformatic analysis was conducted using OECloud tools (https://cloud.oebiotech.com, accessed on 17 September 2023). Principal component analysis (PCA) and orthogonal partial least squares-discriminant analysis (PLS-DA) were used for differential metabolite analysis. The quality of the model was described using the R2X (PCA) or R2Y (PLS-DA) and Q2 values. R2X and R2Y, the proportions of variance in the data explained using the models, indicated the goodness of fit. Q2, the proportion of variance in the data predicted using the model, was calculated using the cross-validation procedure and functioned as an indicator of the predictability of the current model. To avoid model overfitting, a default 7-round cross-validation was performed to determine the optimal number of principal components. A combination of univariate and multivariate analyses was used to screen for differential metabolites. The variable importance for projection (VIP) of the PLS-DA model was used to measure the strength of influence and explanatory power of the expression pattern of each metabolite, thus assisting in the screening of metabolites. Student’s t test was further utilized to verify whether the differential metabolites between groups were significant. The differential metabolites were identified according to VIP values of the PLS-DA model as those greater than 1.5 and with *q* values of univariate statistical analysis lower than 0.01. Fold change was calculated as the average expression ratio between test group and control group, with positive values indicating that the average mass response of the test group was higher than that of control group. The selected screening criteria of fold change (FC) > 1000 and FC > 2.0 were used in the LC-MS and GC–MS analyses, respectively, to identify the main bioactive metabolites.

## Figures and Tables

**Table 1:** Differentially abundant metabolites in LC–MS and their biocontrol effects in previous studies. VIP scores and p-values of each metabolite are also presented.

Classification	Metabolites	VIP	*q* Value	Fold Change	Biocontrol Effects	References
Steroids and steroid derivatives	Telocinobufagin	16.27	2.35 × 10^−3^	3.95 × 10^3^	Fungi, bacteria	[31]
	5beta-cholestan-3alpha,7alpha,12alpha,24(S),27-pentol	3.48	2.25 × 10^−6^	7.22 × 10^4^		
	17a,21-Dihydroxy-5b-pregnane-3,11,20-trione	2.22	9.29 × 10^−6^	3.68 × 10^3^		
	(22E)-12alpha-Hydroxy-3-oxochola-1,4,22-trien-24-oic Acid	2.00	3.27 × 10^−9^	5.14 × 10^3^		
	Chenodeoxycholylglutamine	1.92	6.88 × 10^−5^	6.68 × 10^4^		
Fatty acyls	Macrolactin C	5.02	9.05 × 10^−4^	3.41 × 10^3^		
	Mepartricin B	4.36	2.65 × 10^−5^	2.89 × 10^4^	Fungi	[32]
	cis-tetradec-11-enoic acid	2.78	3.83 × 10^−4^	1.01 × 10^3^		
	N-Palmitoyl glutamine	2.16	1.17 × 10^−4^	4.73 × 10^4^		
	MG (18:2(9Z,12Z)/0:0/0:0)	1.81	1.80 × 10^−4^	2.44 × 10^3^		
Macrolides	7-O-Succinyl macrolactin A	17.87	3.33 × 10^−9^	1.49 × 10^4^	Fungi, bacteria	[33,34]
	Deforolimus	1.51	2.83 × 10^−5^	1.51 × 10^4^		
Organooxygen compounds	Tunicamycin A	2.27	2.61 × 10^−3^	1.69 × 10^10^	Bacteria	[35]
	Macrolactin B	1.59	4.08 × 10^−7^	3.05 × 10^3^	Bacteria	[36]
Glycerophospholipids	CDP-DG (16:1(9Z)/22:5(7Z,10Z,13Z,16Z,19Z))	2.35	1.13 × 10^−3^	3.56 × 10^4^		
	OOV-PS	2.29	2.68 × 10^−5^	4.42 × 10^3^		
Peptidomimetics	Surfactin A	14.79	1.20 × 10^−3^	3.69 × 10^3^	Fungi, bacteria, virus, Mycoplasma, biofilm, induction of ISR	[17,37]
Prenol lipids	Goshonoside F1	2.85	2.06 × 10^−8^	4.67 × 10^3^		
Polyketides	Armochaetoglasin K	1.94	1.22 × 10^−3^	1.61 × 10^3^		

**Table 2:** Differentially abundant metabolites in GC–MS and their biocontrol effects in previous studies. VIP scores and *p*-values of each metabolite are also presented.

Classification	Metabolites	VIP	*q* Value	Fold Change	Biocontrol Effects	References
Organonitrogen compounds	Putrescine	2.17	1.95 × 10^−5^	1.06 × 10^2^	Fungi, plant growth, abiotic stress responses	[38,39,40]
	Spermidine	1.63	4.00 × 10^−6^	1.40 × 10^1^	Plant growth, abiotic stress responses, biofilm	[38,39]
	Ethanolamine	1.23	5.42 × 10^−3^	4.78 × 10^0^		
	Cadaverine	1.01	1.54 × 10^−3^	2.86 × 10^0^	Plant growth, stress response, insect	[41]
Fatty acyls	Heptadecan-1-Ol	1.94	8.09 × 10^−5^	4.17 × 10^1^		
	4-Hydroxybutyric acid	1.60	1.90 × 10^−3^	1.19 × 10^1^		
	Margaric acid	1.57	6.23 × 10^−4^	1.04 × 10^1^		
	Caproic acid	1.19	5.92 × 10^−4^	4.15 × 10^0^	Fungi	[42]
Organooxygen compounds	2,3-Butanediol	1.81	2.31 × 10^−6^	2.58 × 10^1^	Induction of ISR, plant growth, bacteria, fungi	[17,43]
	D-Arabitol	1.32	2.88 × 10^−6^	5.62 × 10^0^		
Imidazopyrimidines	2-Hydroxyadenine	1.34	9.87 × 10^−3^	6.43 × 10^0^		
	Hypoxanthine	1.27	2.30 × 10^−2^	5.60 × 10^0^		
Benzene and substituted derivatives	Phenylboronic acid	2.43	3.29 × 10^−4^	3.39 × 10^2^		
Keto acids and derivatives	Acetoacetic acid	1.70	1.03 × 10^−4^	1.76 × 10^1^	Biofilm, bacteria	[44]
Organic carbonic acids and derivatives	Urea	1.54	2.72 × 10^−2^	1.13 × 10^1^	Fungi, bacteria	[45]
Purine nucleotides	Inosinic acid	1.49	5.99 × 10^−3^	7.42 × 10^0^		
Organic phosphoric acids and derivatives	Methylphosphate	1.49	1.56 × 10^−5^	9.00 × 10^0^		
Diazines	Dihydrouracil	1.44	4.57 × 10^−4^	7.94 × 10^0^	Nematodes	[46]
Pteridines and derivatives	Pterin	1.36	1.07 × 10^−3^	6.36 × 10^0^	biofilm, bacteria	[47]
Purine nucleotides	Adenosine monophosphate	1.08	2.05 × 10^−2^	3.17 × 10^0^		
Unclassified	5-Methoxy-1-Pentanol	2.19	5.54 × 10^−3^	1.22 × 10^2^		
	Hydrobenzoin	1.25	3.66 × 10^−6^	4.68 × 10^0^		
	4-Nitrophenethylamine	1.24	1.19 × 10^−4^	4.66 × 10^0^		

## Data Availability

All raw sequence data reads are available at the NCBI SRA under accession number SRR25593771-SRR25593785 (BioProject accession number PRJNA1004066 for Bacteria).

## Supplementary Materials

The following supporting information can be downloaded at: https://www.mdpi.com/article/10.3390/ijms242316694/s1.
