# Supplementary material for: Antifungal Effect of Bacillus velezensis ZN-S10 against Plant Pathogen Colletotrichum changpingense and Its Inhibition Mechanism
Source: Int J Mol Sci. 2023 Nov 24;24(23):16694. doi: 10.3390/ijms242316694 (PMC10705930; doi:10.3390/ijms242316694)
Supplement: Supplementary file 1 [file ijms-24-16694-s001.zip › ijms-2676752-supplementary.pdf]

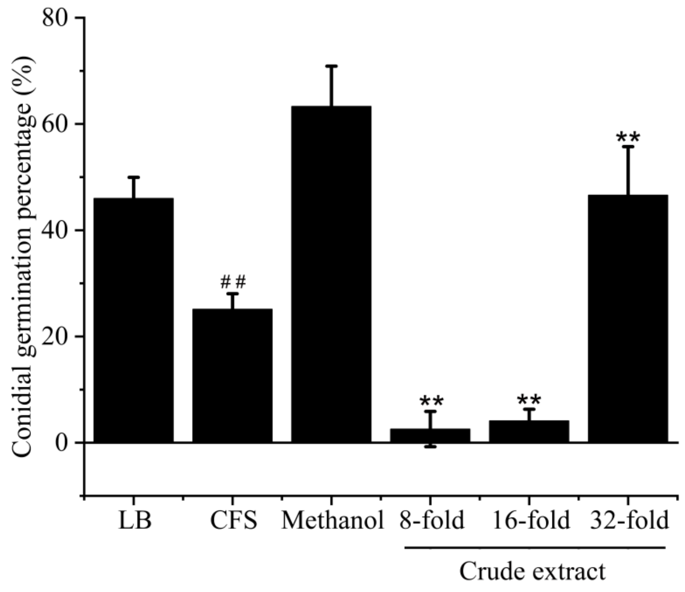

Figure S1. Percentage of conidial germination of *C. changpingense* ZAFU0163-1 in the presence of cell-free synthesis (CFS) and C18 solid phase extraction (SPE) crude extract of *B. velezensis* ZN-S10. Asterisks represent differences compared with methanol (**p ≤ 0.01); compared with LB (##p ≤ 0.01).

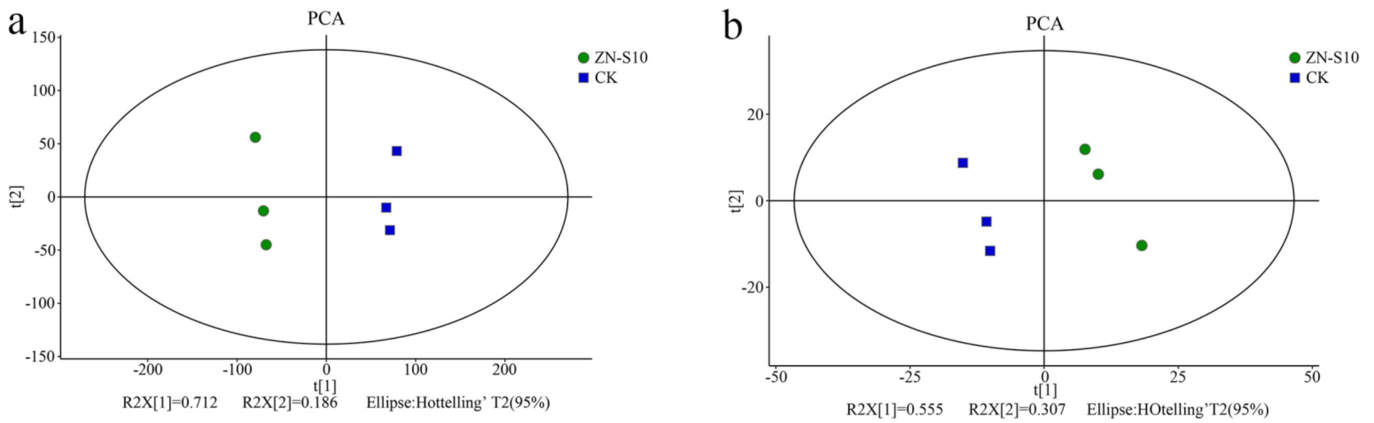

Figure S2. PCA score plots of (a) LC–MS and (b) GC–MS metabolite profiles. t[1] and t[2] denote the score value of each sample projected on the principal components PC1 and PC2, respectively. The elliptical regions represent 95% confidence intervals.

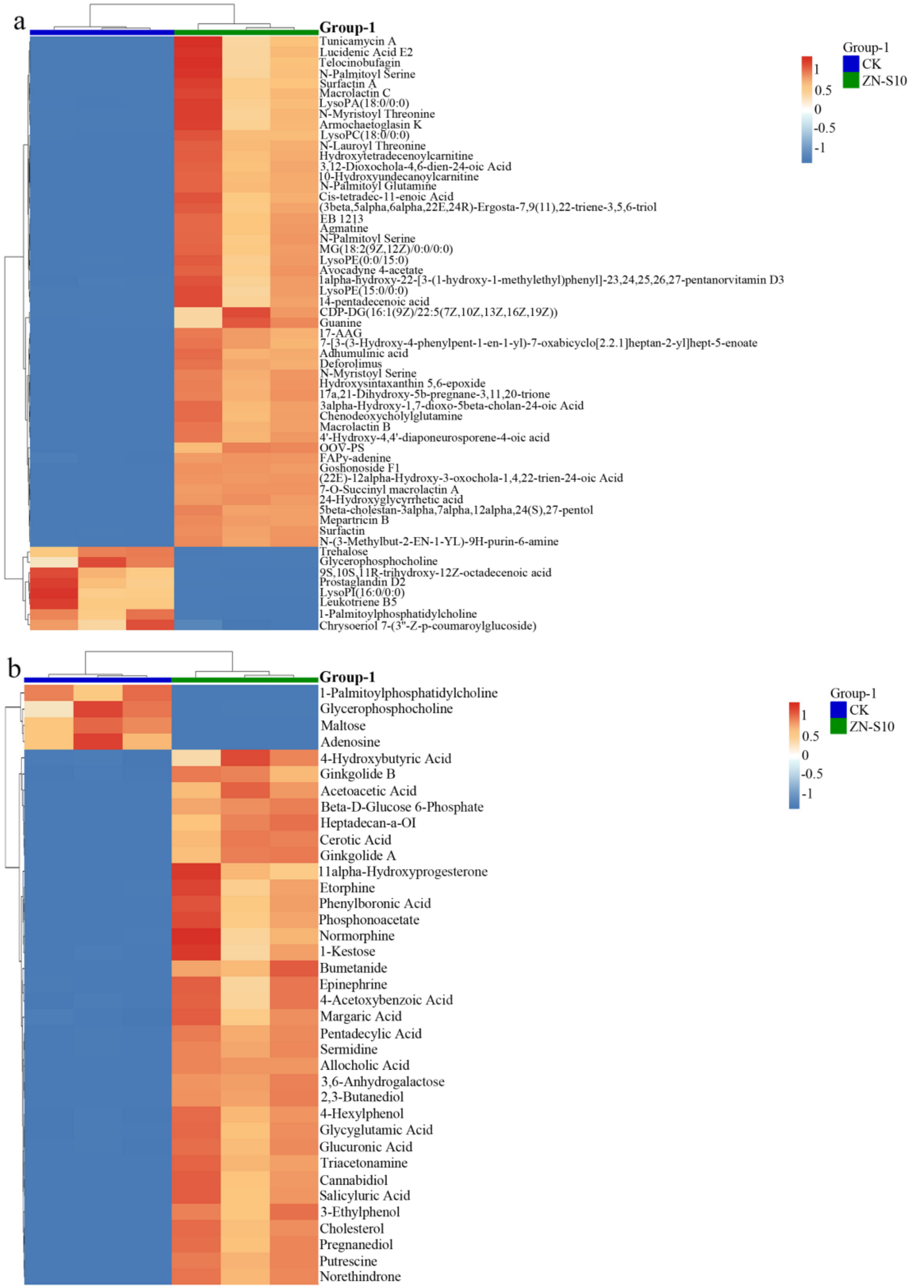

Figure S3. Heatmap analysis of the differential metabolites in (a) LC–MS, (b) GC–MS. The tree indicates the similarity of metabolites. The color from blue to red indicates that the expression abundance of the metabolite is from low to high, the redder the metabolite, the higher the expression abundance of the differential metabolite, and each column represents one sample (VIP values > 1.5 and q values < 0.01).
